# A meta-analysis on the heavy metal uptake in *Amaranthus* species

**DOI:** 10.1007/s11356-023-28374-3

**Published:** 2023-06-28

**Authors:** Dávid Tőzsér, Ayash Yelamanova, Bianka Sipos, Tibor Magura, Edina Simon

**Affiliations:** 1 Department of Ecology, University of Debrecen, Egyetem Sq. 1, 4032 Debrecen, Hungary; 2 Circular Economy Analysis Center, Hungarian University of Agriculture and Life Sciences, Páter Károly str. 1, H-2100 Gödöllő, Hungary; 3 ELKH-DE Anthropocene Ecology Research Group, Egyetem Tér 1, 4032 Debrecen, Hungary

**Keywords:** Amaranth, Phytoremediation, Bioaccumulation, Relative interaction intensity, BAF, Web of Science

## Abstract

Metals can accumulate in different parts of plant species in high concentrations, which gives the basis for the plant-based technology called phytoremediation. Among annual species, *Amaranthus* is a well-studied, potential metal accumulator genus; however, some conflicts are found among published results. Thus, we studied the metal (Cd, Cu, Fe, Ni, Pb, and Zn) accumulation potential of *Amaranthus* plant parts (root, stem, and leaf) by meta-analysis, furthermore, by calculation of bioaccumulation factor (BAF) values. After the extensive literature search and the calculation of relative interaction intensity (RII) values, we found significant accumulation for each metal by *Amaranthus* individuals growing on contaminated soils compared to plants collected from uncontaminated ones. Differences among plant parts were significant for Cu and Fe, minor for Ni, Pb, and Zn, and negligible for Cd. The BAF values indicated high accumulation in the leaf, moderate in root and stem for Cd, moderate in each plant part for Pb, and very low in each plant part for Fe, Ni, and Zn. We highlight that *Amaranthus* species are good prospects for metal phytoremediation projects, although, due to specific plant part-metal patterns, special attention should be paid to the harvesting practice.

**Supplementary Information:**

The online version contains supplementary material available at 10.1007/s11356-023-28374-3.

## Introduction

Recently, issues related to soil contamination have been given more attention worldwide. Contaminated soils usually result from past processes when impacts associated with the fabrication, usage, and removal of hazardous substances were less known than today (Shang et al. 2019). Therefore, over the decades, the issue of soil contamination has become global, while the number and extent of the area of affected sites are vast (Panagos et al. 2013; Bech 2022). Furthermore, in case these contaminants get into the food web, they can also threaten nutritional security, aquatic resources, rural livelihoods, and human well-being (Gall et al. 2015).

Among contaminants, the presence of toxic metals in soil can adversely affect organism-mediated soil processes because metals decrease the amount of soil microbial biomass (Briffa et al. 2020). Despite most of the metals being vital micronutrients for plants, animals, and humans, their high concentration could be a reason for phytotoxicity and various human disease due to their accumulation in the tissues of living organisms (FAO 2018). Moreover, contaminated biomass poses potential harm to the environment and the food chain (Rai et al. 2019). In addition, a high concentration of toxic elements in soil reduces plant metabolism and has a serious effect on crop yield, exerting pressure on cultivated lands. Despite the negative impacts of heavy metals on the health of ecosystems affected, the presence of heavy metals in some natural environments has led to the evolution of plants with the ability to resist, tolerate, or even thrive on metalliferous soils (Ali et al. 2013; Rajakaruna et al. 2014).

To remove metals from the soil, it is reasonable to set up an environmentally sound and sustainable technique, which can be carried out in situ (on-the-site treatment) or ex situ (treatment away from the contaminated site) (Wadgaonkar et al. 2019). Employable techniques can be classified as physical, chemical, and biological practices, suggesting possible technical solutions to most soil contamination types (Sharma et al. 2018). Plant vegetative organs can serve as perfect agents for soil remediation due to their natural ability to accumulate metals from the soil based on their unique genetic, biochemical, and physiological traits (Ziarati and Somaye Alaedini 2014; Suman et al. 2018). Thus, remediation with plants remains cost-effective, with only minor costs arising during installation and maintenance, such as weed control (Hauptvogl et al. 2020). Furthermore, the involvement of plants provides several advantages; the use of plant biomass as a basis for heat and energy production, and— in case of high tissue concentration— the potentially extractable quantity of metals can make the technology profitable (Abhilash and Yunus 2011; Yan et al. 2020).

Several hundred weeds have been proven to be good candidates for phytoremediation purposes due to their ability to decrease metal concentration in soil; these species are stress-tolerant, usually with fast growth and biomass production enabling even in a wide variety of environments (Tőzsér et al. 2019; Alizadeh et al. 2021; Melo et al. 2022). These characteristics can also be observed in several *Amaranthus* species. *Amaranthus* species are found in the Caryophyllales order, Amaranthaceae family, Amaranthoideae subfamily, and *Amaranthus* genus which includes about 43 species in Europe (Wolosik and Markowska 2019; Iamonico 2020). Additionally, they are spread in many parts of the world like Central and South America, Africa, China, India, and the USA, which makes them viable options to be involved in remediation studies (Wolosik and Markowska 2019; Aguilera-Cauich et al. 2021). Indicating the diversity of relevant studies, the accumulation of heavy metals by *Amaranthus* spp. has been recently investigated at garbage dumpsites, animal waste dumpsites, and single- and multi-element-contaminated soils (Chunilall et al. 2005; Adefila et al. 2010; Adefemi et al. 2012; Akubugwo et al. 2012; Shagal et al. 2012). For example, *Amaranthus caudatus* L. plants grown on moderately multi-contaminated soils were indicated to accumulate a high concentration of metals, including Cd, Cu, Fe, Mn, and Pb (Adewuyi et al. 2010). Nevertheless, relevant results on metal accumulation in *Amaranthus* are quite contradictory. It is in strong relation with the fact that the genus features more than a hundred species having quite various individual characteristics, i.e., remediation capabilities, which is highlighted even more by the influence of external factors (e.g. moisture content, pH, structure, and single/multi-contamination of the soil, bioavailable metal pool). For instance, Yap et al. (2022) showed very low Cd accumulation in *Amaranthus* leaves, while according to Fan and Zhou (2009), the accumulation rate was higher than the threshold values for hyperaccumulation. Additionally, the accumulation rate of Pb was reported to be high in shoots by Rahman et al. (2013), while that was found much lower by Oluwatosin et al. (2010), and by Yap et al. (2022). These previous results inspire the need for our integrating analyses.

The aim of this paper was to analyze the accumulations of Cd, Cu, Fe, Ni, Pb, and Zn—the most frequently studied metals in this respect—among selected plant parts (root, stem, and leaf) of *Amaranthus* spp. growing in contaminated soils, using literature-based meta-analysis. Based on the results of earlier papers, significantly more intensive accumulation was hypothesized in *Amaranthus* grown on contaminated soils than ones from uncontaminated soils. We also hypothesized that metals accumulate in each plant part, however, in different concentrations; we assumed Pb and Cd to accumulate primarily in roots, while Cu, Fe, Ni, and Zn were expected to reach the highest concentrations in aboveground plant parts, with high bioaccumulation (BAF) values for above metal-plant part relations.

## Materials and methods

### Literature search

We performed a literature search on the Web of Science for the period 1975–2022, based on the following search terms: TOPIC = (*Amaranthus*) and TOPIC = (*metal* OR *phytoremediation*). In addition, we studied the reference section of the publications found in this search for extra, undiscovered, corresponding papers. To be incorporated, a paper had to report metal (Cd, Cu, Fe, Ni, Pb, and/or Zn) concentration in plant parts (root, and/or stem, and/or leaf) of *Amaranthus* spp. growing in contaminated vs. uncontaminated soils; contaminated and uncontaminated soils were determined according to the categorization by the authors of the papers involved. Data were retrieved from text, tables, and graphs. As we aimed to evaluate the inherent phytoextraction potential of *Amaranthus* spp. publications, in which any compound (e.g., EDTA) had been applied to foster metal absorption were excluded from the assessment.

### Statistical analyses

The effect size of metal accumulation for each uncontaminated-to-contaminated comparison was calculated using the unstandardized mean difference (relative interaction intensity, RII, Armas et al. 2004). RII is defined as follows:

1 $$RII=\frac{\overline{{X }_{U}}-\overline{{X }_{C}}}{\overline{{X }_{U}}+\overline{{X }_{C}}}$$

where $\overline{{X }_{U}}$ and $\overline{{X }_{C}}$ are the mean metal concentration (mg kg^−1^, dry matter) in plant parts of *Amaranthus* spp. growing in uncontaminated (*U*) and contaminated (*C*) soils.

Relative interaction intensity was used because only 10% of the datasets presented both variance and sample size, which are needed to calculate the standardized mean difference, while RII calculation can be done by including mean values alone. For this reason, studies without reporting variance, but meeting all the other criteria, could be involved in this analysis. Negative values indicate higher metal concentration in *Amaranthus* plant parts growing in contaminated soils than in uncontaminated ones, while positive values indicate the opposite. RII values were calculated for each selected plant part-metal pair if the sample size (*n*) was ≥ 5. The relative interaction intensity with bootstrapped confidence intervals (with 9,999 iterations) was calculated using the *boot* package (Davison and Hinkley 1997; Canty and Ripley 2015). Where the confidence intervals did not include zero, we considered the effect size to be significantly different from zero.

To assess the metal accumulation potential in *Amaranthus* more precisely, the degree of accumulation was assessed by the calculation of bioaccumulation factor (BAF) values, based on the soil and plant part concentration values extracted from the publications:

2 $$BAF=\frac{{C}_{\mathrm{plant part}}}{{C}_{\mathrm{soil}}}$$

where *C*_plant part_ is the metal concentration (mg kg^−1^, dry matter) measured in the selected plant part, and *C*_soil_ is the metal concentration (mg kg^−1^, dry matter) measured in the growing media.

All analyses were conducted using R version 4.1.2 (R Development Core and Team 2011).

## Results

**Fig. 1:**
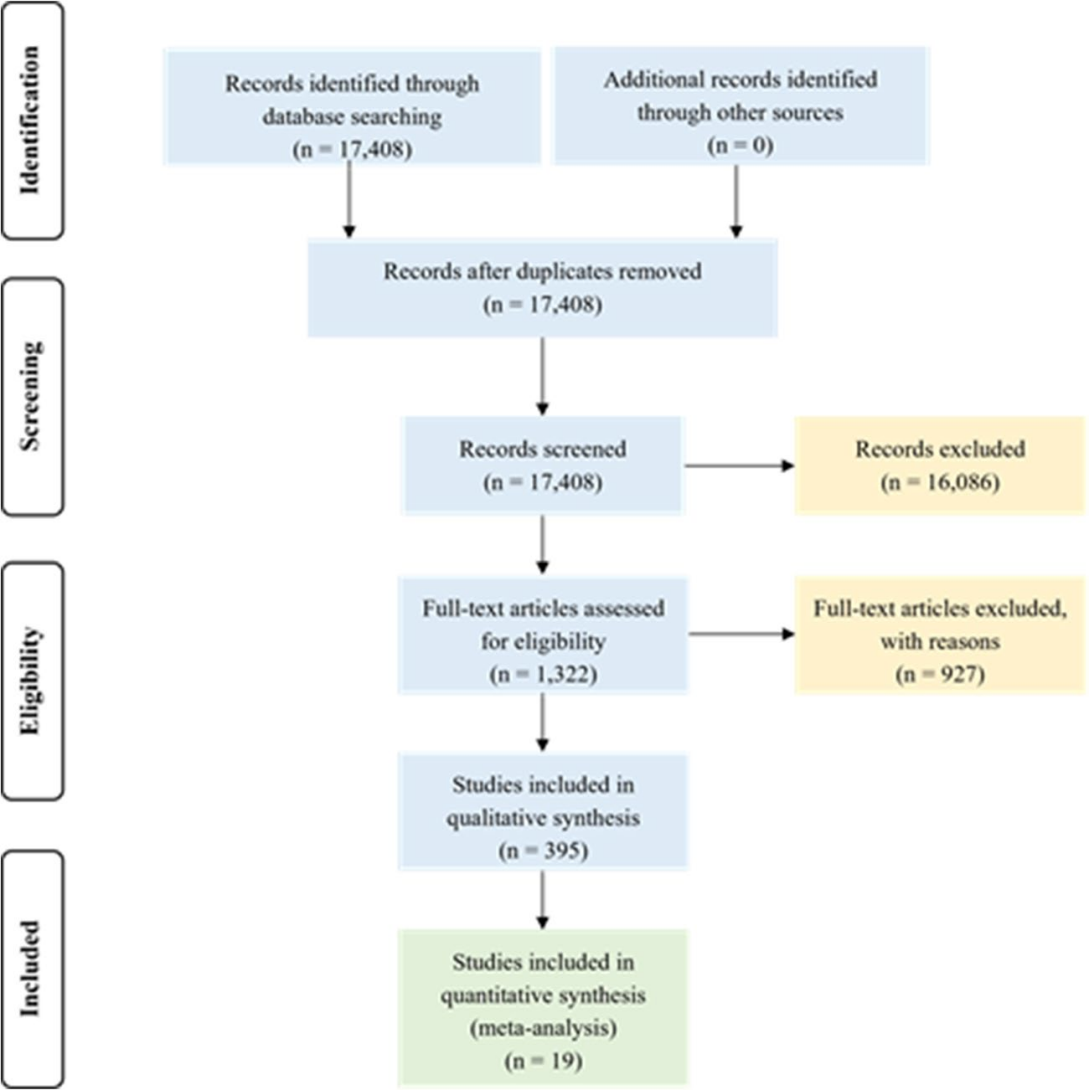
PRISMA flow diagram of the selection process through the analysis (i.e., the number of studies identified, excluded, and included)

The Web of Science-based literature search yielded a total number of 17,408 articles. Out of these publications, 19 papers fulfilled the criteria set for the meta-analyses (Fig. 1, Table 1, Supplementary Information Table 1). From these papers, 332 uncontaminated-contaminated comparisons were extracted. Contaminated soil metal concentrations presented in the selected papers were wide-ranging: 0.3–200 mg kg^−1^ for Cd, 13.5–3480 mg kg^−1^ for Cu, 6.08–19,254 mg kg^−1^ for Fe, 2.50–100 mg kg^−1^ for Ni, 3.00–151 mg kg^−1^ for Pb, and 14.0–819.5 mg kg^−1^ for Zn.

**Table 1:** Data of papers involved in the meta-analyses of *Amaranthus* species

Authors	Studied species	Studied plant part	Studied metals	Experimental location	Type of experiment	Number of comparisons
Alsherif et al. (2022)	*A. retroflexus*	Root	Cd, Cu, Fe, Ni, Pb, Zn	Khulais, Saudi Arabia	Field	24
Atayase et al. (2008)	*A. viridis*	Root, stem, leaf	Cd, Pb	Lagos, Nigeria	Field	48
Bosiacki et al. (2013)	*A. caudatus*	Stem, leaf	Cd, Pb	Poznan, Poland	Greenhouse	12
Chinmayee et al. (2012)	*A. spinosus*	Root, stem, leaf	Cd, Cu, Zn	Kerala, India	Greenhouse	27
Chunilall et al. (2005)	*A. dubius*, *A. hybridus*	Root, stem, leaf	Cd, Ni, Pb	KwaZulu-Natal, South Africa	Greenhouse	54
Cui et al. (2021)	*A. hypocondriacus*	Root	Cd	Shaoguan, China	Greenhouse	5
Ding et al. (2013)	*A. hypocondriacus*	Root, stem, leaf	Cd	Guangzhou, China	Greenhouse	12
Egwu et al. (2019)	*A. cruentus*	Leaf	Cd, Pb	Chanchaga, Nigeria	Field	2
Eze (2014)	*A. hybridus*	Root, stem, leaf	Cd, Fe, Ni, Pb, Zn	Gombe, Nigeria	Field	56
Garba and Kiyawa (2018)	*A. hybridus*	Root, stem, leaf	Cu, Fe, Ni	Kano, Nigeria	Greenhouse	7
Ghazaryan et al. (2021)	*A. retroflexus*	Root	Cu	Kajaran, Armenia	Pot (ex situ)	1
Huang et al. (2019)	*A. spinosus*	Root	Cd, Pb	Yichang, China	Greenhouse	8
Khoramnejadian and Saeb (2015)	*A. retroflexus*	Root	Cd, Cu, Ni	Damavand, Iran	Pot (ex situ)	3
Liu et al. (2019)	*A. retroflexus*	Root, stem, leaf	Cu	Taiyuan, China	Greenhouse	9
Liu et al. (2021)	*A. tricolor*	Root, stem, leaf	Cd	Shaoguan, China	Greenhouse	24
Motesharezadeh et al. (2010)	*A. retroflexus*	Root	Cd	Karaj, Iran	Greenhouse	3
Nejatzadeh-Barandozi and Gholami-Borujeni (2014)	*A. retroflexus*	Root	Cd, Pb	Urmia, Azerbaijan	Field	10
Ramírez et al. (2021)	*A. dubius*	Root	Pb, Zn	Haina, Santo Domingo	Field	20
Zou et al. (2006)	*A. viridis*	Root, stem, leaf	Cu, Fe, Zn	Tianjin, China	Field	7

### Meta-analysis of the metal accumulation in *Amaranthus*

**Fig. 2:**
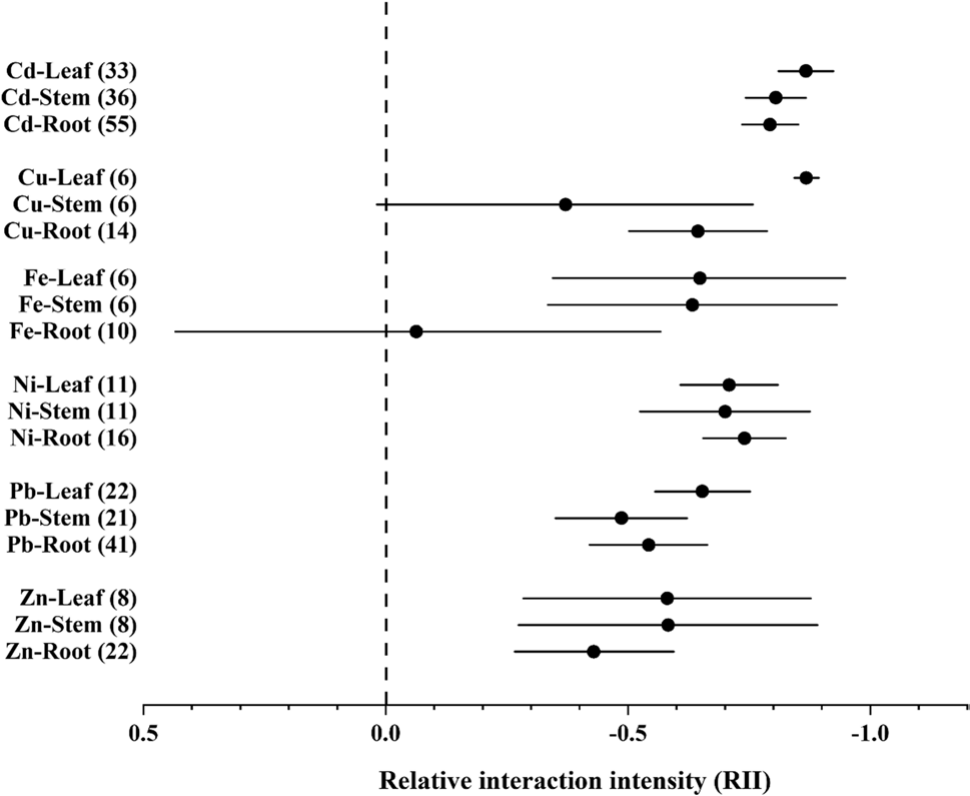
Relative interaction intensity (RII) values for Cd, Cu, Fe, Ni, Pb, and Zn accumulations in plant parts of *Amaranthus* (with the number of comparisons in brackets)

Each plant part (root, stem, and leaf) of *Amaranthus* spp. accumulated Cd, Ni, Pb, and Zn in significantly higher concentrations on contaminated soils than control individuals growing on uncontaminated soils (RII values below null; 95% confidence interval did not reach null; Fig. 2). The accumulation level was low for Cu in the stem and Fe in the root, with significant accumulation in the other plant organs for both metals. Besides afore two plant organ-metal relations, significant differences were not found in the accumulation of metals among plant organs. In general, the accumulation of Cd was the most intensive among all the metals studied (Fig. 2).

### Bioaccumulation factors (BAF) for metals in plant parts of *Amaranthus*

**Fig. 3:**
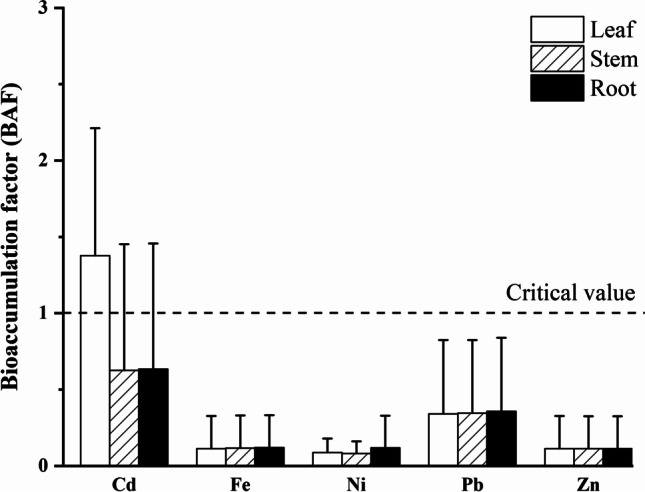
Bioaccumulation factor (BAF) values (mean ± SD) for *Amaranthus* spp. growing on contaminated soils. (Number of studies and data: Cd: 7 studies, *N* = 47; Fe: 4 studies, *N* = 22; Ni: 4 studies, *N* = 20; Pb: 5 studies, *N* = 45; Zn: 3 studies, *N* = 19.) Values greater than the critical value (> 1) indicate intensive accumulation of metals from the soil

According to BAF values, the accumulation potential for Cd in the leaf was high (> 1 = critical value), since Cd concentration in the leaf was higher than that in the soil. In general, Cd accumulation was high in the leaf and moderate in the root and stem of *Amaranthus* spp. This suggests the studied species’ great ability to translocate Cd into aboveground plant parts (leaf >  > stem≊root; Fig. 3). Bioaccumulation was of low intensity for Pb in each plant part with lower concentrations in tissues than in soils; BAF values for leaf and stem were lower than the critical value. Furthermore, there was no difference in Pb accumulation among plant parts (root≊stem≊leaf; Fig. 3). BAF values indicated that the accumulation potential for Fe, Ni, and Zn was lower than the critical value in each plant part, since all the tissues contained metals in much lower concentrations than that in the soil; additionally, accumulation preferences for metals in plant parts were the following: root≊stem≊leaf for Fe, root≊stem < leaf for Ni, root≊stem≊leaf for Zn (Fig. 3). (Sample size for Cu was not enough for the BAF analyses.)

## Discussion

We demonstrated that heavy metals accumulated in plant parts of *Amaranthus* spp. in different concentrations. It was supported by the relative interaction intensity (RII) values (uncontaminated-contaminated comparison) that the accumulation of Cd, Cu, Fe, Ni, Pb, and Zn was significant in *Amaranthus*, showing metal-dependent differences in intensity among plant parts. Furthermore, our study on bioaccumulation factor (BAF) values (contaminated soil-contaminated plant comparison) revealed that Cd was accumulated primarily in leaves, while in cases of Cu, Fe, Ni, Pb, and Zn, there were no major differences in metal concentrations among plant parts. Additionally, results should be interpreted the potentially accessible data, the presence and lack of which are practically highlighted first.

### Limitation of data

Besides having adequate data for the analyses, several factors influencing metal accumulation in *Amaranthus* could only scarcely be assessed. Without a complete overview, here, some of the most relevant variables were highlighted in terms of their data availability among included publications. Within *Amaranthus*, the metal accumulation of species is widely different, while, in general, that of populations of the same species from different geographical locations can also vary (Ranđelović et al. 2018; Ramanlal et al. 2020). In this paper, however, sample sizes for metal-plant part relations in individual species did not enable comparisons by species, while the relevancy of studying the effect of location was low because only 37% of the publications conducted the tests on the field. As well, contamination schemes (low/moderate/high and single/multi-contamination) are major influentials by plants’ metal accumulation (Shojaei et al. 2021). In this study, the limitation was caused by the fact that only 37% of the papers assessed highly contaminated soils, and only 26% of the publications reported data from single-contaminated growing media. For extensive evaluations, soil properties and their influences should be considered too (Xu et al. 2022). The remediation efficiency of plants due to the bioavailable pool of metals is fundamental (Ng et al. 2015). Most of the comparisons (80%) involved in this study were based on total soil concentrations, thereby the integration of bioavailable content into the analyses was not considered favorable. In the analyses, 65% of the papers assessed basic soil properties (soil type and/or pH and/or soil organic matter content), however, studied parameters were too diverse to get desired sample sizes with only 26% of these from the field. This latter also hindered the reliable evaluation of another core factor in plant growth and metal accumulation, namely the date (season) of sampling (Devlaeminck et al. 2004); the majority of the papers conducted tests under a controlled (pot/greenhouse) environment, which masks the effects of sampling season.

### Cd accumulation in *Amaranthus*

Analyzing the RII values, we found that the accumulation of Cd was significant in *Amaranthus*, with similar intensities among plant parts. Furthermore, evaluating the bioaccumulation factor (BAF) values, we found that the leaf is a very good Cd accumulator tissue. In their study, Antonkiewicz and Jasiewicz (2002) reported that the concentration of Cd in five species, including *Amaranthus hybridus* E.H.L.Krause was significantly higher in aboveground plant parts than in roots. Contrarily, studying two *Amaranthus mangostanus* L. cultivars (Chi et al. 2019), the root was proved as the primary Cd depository also by increased contamination levels in soil, finding the storage capacity of intercellular spaces, cell walls, and root apoplast as the main reason behind. In the cases of other herbaceous species, however, leaf Cd accumulation is a frequent phenomenon, which suggests a wide interspecific variation in the pattern of metal accumulation (Lombi et al. 2001; Baldantoni et al. 2016; Ullah et al. 2020). We also demonstrated a high accumulation potential of the leaf of *Amaranthus* species growing in contaminated soils (BAF > 1). In the literature, reported BAF values for *Amaranthus* and other herbaceous genera usually oppose our findings. Among others, Adefila et al. (2010) found low accumulation rates in leaves compared to roots of *Amaranthus viridis* L. individuals. Additionally, El-Amier et al. (2017) demonstrated generally low Cd BAF values for aboveground plant parts of six weeds, while those for roots neared or exceeded 1 in each species. Although Cd tends not to accumulate in aboveground plant parts in high concentrations (Aladesanmi et al. 2019), we highlight *Amaranthus* as a prospect to extract Cd also by storing the metal in harvestable plant parts. As potential reasons for the above inconsistencies in Cd accumulation, physicochemical parameters (e.g., interactions among metals triggered by their simultaneous presence in soil, substitution by accumulation) and habitat factors (e.g., soil pH and moisture conditions, exposure time, plant interspecific competition) can also be listed (Tőzsér et al. 2017; Huang et al. 2020).

### Cu accumulation in *Amaranthus*

We demonstrated highly plant part-specific accumulation for Cu in *Amaranthus* with the leaf as the most intensive accumulator and the stem as the least intensive accumulator tissue. In contrast with our findings, Mellem et al. (2012) demonstrated the roots as the main Cu-depository plant parts of *Amaranthus dubius* Mart.; therefore, its remediation potential was limited. The same conclusion was drawn also by Ogunkunle et al. (2013), who found interspecific differences in tolerance to be the main reasons for the retention of the majority of Cu in roots. As another factor potentially affecting (hindering) Cu accumulation, Prudent et al. (2014) highlighted the basic role of metal interactions when assessing relevant patterns in *Amaranthus*. After studying six leafy vegetables out of which two were *Amaranthus lividus* L. and *Amaranthus gangeticus* L., Ahmed et al. (2022) found the lowest Cu concentrations for the two *Amaranthus* species. Furthermore, soil amendments (e.g., S,S-EDDS) were found especially effective in solubilizing Cu and triggering its translocation into the leaves of *A. viridis* and *A. caudatus* (Ko et al. 2013). We observed this pattern without the use of any substances, indicating the effectiveness of *Amaranthus* leaves in Cu accumulation based on its inherent ability. Based on these afore, we assume that contrasting results can be linked to the metal interactions and specific variances in Cu tolerance, which could be supported by the extensive statistical comparison of individual *Amaranthus* species in this regard.

### Fe accumulation in *Amaranthus*

Using the meta-analytical method, we found that the accumulation of Fe was significant in *Amaranthus*, with similar intensities among plant parts. Furthermore, evaluating bioaccumulation factor (BAF) values, we showed that accumulation of Fe was similarly very low in and among plant parts. Relevant information on Fe accumulation in *Amaranthus* spp. was published only in those papers, on which our meta-analysis was based. Therefore, our result should be interpreted by focusing on other herbaceous genera. By doing so, we observe several inconsistencies. Similar to our results, El-Amier et al. (2017) indicated very low bioaccumulation in all the plant parts for all the six studied species, not favoring their use for the phytoextraction of Fe. Contrarily, Boamponsem et al. (2012) reported that Fe was accumulated in considerably higher concentration in roots than in other plant parts of *Brassica*. Here, the authors also observed the effect of Fe on the accumulation of other metals, as a potential explanation for the pattern; the low content of Cd in *Brassica oleracea* L. var. capitata and *Lactuca sativa* L. could be an impact of Fe presence in plants; the authors found that excess Fe reduced the absorption of Cd. Sharma et al. (2004) also reported an antagonistic relationship between Cd and Fe during the accumulation. Although we found high-intensity Fe accumulation in contaminated sites compared to control ones, reduced accumulation from soils (very low BAF values) can be explained by high simultaneous Cd concentrations. Furthermore, Freitas et al. (2015) provided evidence that some of the microorganisms release organic substances, which enhance bioavailability and increased root absorption of essential metals like Fe, while others can hinder the accumulation of such elements. The microbial composition of soils is not investigated in the publications we included, but the presumed effects of these communities on reduced Fe accumulation should not be excluded from the scope as potential factors.

### Ni accumulation in *Amaranthus*

We found by meta-analyses that the accumulation of Ni was significant in *Amaranthus*, with slightly different intensities among plant parts. Furthermore, we reported using the bioaccumulation factor (BAF) values that the accumulation of Ni was similarly very low in and among plant parts. In our meta-analysis, we found that root accumulation was the most intensive, which was not supported by the very low BAF values in each plant part. Chunilall et al. (2005) reported similar conclusions about *Amaranthus* and identified the root as the most intensively accumulating plant part; however, the authors found higher differences in the accumulation potential than we did, mentioning *Amaranthus* as excluders for Ni. As for other genera, Berton et al. (2006) reported Ni concentration in *Phaseolus vulgaris* L. grown in highly Ni-contaminated soil exceeding the maximum tolerable threshold value (5 mg kg^−1^), with the highest concentration found also in the root. This latter was also noticed for *Fragaria ananassa* Duchesne in a study by Roveda et al. (2016). Studying the maze, Lu et al. (2015) detected the highest concentration of Ni in roots; the authors highlighted that a great amount of Ni was accumulated in plant roots, despite the low soil Ni concentration. Also similarly to our results, Lago-Vila et al. (2015) emphasized the role of roots in accumulating Ni in high concentrations; therefore, according to the BAF values higher (> 1) than reported by our study, they classified the tested *Festuca* and *Juncus* species as good candidates for Ni phytostabilization. According to the intensive accumulation of individuals growing in Ni-contaminated soil compared to control ones and the low accumulation potential in aboveground plant parts from contaminated soils, we recommend the use of *Amaranthus* for Ni immobilization rather than extraction.

### Pb accumulation in *Amaranthus*

During the meta-analysis, we found that the accumulation of Pb was significant in *Amaranthus*, with slightly different intensities among plant parts. Furthermore, we reported by the bioaccumulation factor (BAF) values that accumulation was similarly low in and among plant parts. In this study, we observed that Pb accumulation in the stem was less intensive than that in the root and leaf, which was not supported by the similarly low BAF values in each plant part. Similar to our results, Abubakar et al. (2014) demonstrated the most intensive Pb accumulation in the root of *A. caudatus*. Furthermore, the authors reported a negative correlation in the concentration between soil and aboveground plant parts, which was linked to the inhibitory characteristics of Pb during its accumulation and translocation into the stem and leaf. Aghelan et al. (2021) also made a similar statement to our results; according to the authors, the root of *A. caudatus* was the best Pb accumulator plant part, which was, however, followed by the stem. Additionally, this latter paper presented lower BAF values for root (< 0.2) and stem (< 0.04) lower than those in this study. Assessing the results for other genera, Amin et al. (2018) also proved root as plant part accumulating Pb in the significantly highest concentrations in *Cyamopsis tetragonoloba* (L.) Taub. and *Sesamum indicum* L. individuals, both of which, unlike *Amaranthus* in this study, were recognized as suggested species in Pb phytoremediation due to their high root BAF values (> 1) and high metal concentrations (> 100 mg kg^−1^). In their paper, Hesami et al. (2018) studied the element composition in the root and shoot of 16 species, out of which 10 species concentrated Pb primarily in the root, rather than in aboveground parts. As a Pb-specific reason for the pattern, the high propensity of Pb to bind with organic and/or colloidal substances, thus the solubility of the Pb compound should also be taken into consideration (Chandrasekhar and Ray 2019). Furthermore, Aery and Rana (2007) concluded that Pb had a highly concentration-dependent nature of interaction with both Cd and Zn, with that being synergistic by low and antagonistic by high metal concentrations. Observing various BAF values for these three metals, we support the findings by Aery and Rana (2007), also in the case of *Amaranthus* species. Additionally, Agoramoorthy et al. (2009) attributed the intensive translocation of Pb to the in-planta and ex-planta multi-contamination patterns complemented by the accumulation-favoring positive effects of the water regime.

### Zn accumulation in *Amaranthus*

Analyzing the RII values, we found that the accumulation of Zn was significant in *Amaranthus*, with different intensities among plant parts. Furthermore, assessing the bioaccumulation factor (BAF) values, we reported that accumulation of Zn was similarly very low in and among plant parts. In this study, we showed that the accumulation of Zn in the stem was less intensive than that in the root and leaf, which was, however, not supported by the similarly very low BAF values in each plant part. The lower Zn accumulation potential of the stem compared to the root and leaf was also indicated by Carrión and Mendoza (2019) in *A. hybridus*, which was explained by the efficient regulation of the species to avoid translocation of Zn into aboveground woody plant parts. Similarly, Ogbenna et al. (2019) highlighted the primary role of leaf in Zn accumulation in *A. caudatus*, with significantly lower concentrations in the stem. Excluding seedlings growing in an extremely Zn-contaminated environment, Lukatkin et al. (2021) reported that *Amaranthus retroflexus* L. was a species accumulating the majority of Zn also in the leaf, however, followed by the stem. Considering the Zn accumulation pattern in other weeds, mainly contrasting conclusions can be seen. Among others, Hesami et al. (2018) indicated various accumulation patterns among studied species, with generally the highest Zn concentrations in the root. Oladejo et al. (2017) demonstrated the same conclusion by assessing maize individuals on Zn-contaminated sites. Studying several terrestrial species, Oti (2015) demonstrated BAF values (< 0.2) similar to those shown in our study. Bioaccumulation was also very restricted (BAF < 0.4) in all the herbaceous species monitored by Rehman et al. (2018), who listed local climate and plant development stages as influencing factors not favoring Zn accumulation. As another aspect, Yang et al. (2020) indicated the role of Cd-Zn antagonism by plant accumulation; with low Zn and high Cd BAF values, we supported the same conclusion. Evaluating these results, we emphasize the ability of *Amaranthus* to be involved in Zn phytoremediation, which, unlike in several other genera, can be successful also by harvesting leaf as a major Zn-depository.

## Conclusions

Based on the results of the meta-analysis, we highlighted that *Amaranthus* spp. accumulated each studied metal in significantly higher concentrations in contaminated soils than in control ones. *Amaranthus* individuals, however, showed significant plant part-based differences in the accumulation of Cu and Fe, while that for Ni, Pb, and Zn was minor. Accumulation differences for Cd were low among root, stem, and leaf. Bioaccumulation factor (BAF) values, comparing the metal concentration in plant parts to metal concentrations in contaminated soils, demonstrated various metal accumulation patterns for plant parts; we found very good Cd accumulation potential in *Amaranthus* leaf, moderate in root and stem, while that for Pb was moderate, for Fe, Ni, and Zn very low in each plant part. Based on the results of the meta-analysis, we conclude that, in general, *Amaranthus* species respond to elevated soil metal concentrations with significant accumulation in their root, stem, and leaf. Furthermore, supported by BAF values, studied amaranth species have a high potential for Cd accumulation, primarily in leaves. Therefore, the collection and harvesting of *Amaranthus* from contaminated sites require the consideration of plant part-specific accumulation patterns, while the involvement of other factors influencing metal accumulation (e.g., bioavailable metal pool, physical soil properties, sampling location) are also keys to perform proper evaluations in future studies.

## Supplementary Information

Below is the link to the electronic supplementary material.

Supplementary file1 (DOCX 412 KB)

## Data Availability

The authors declare that data and materials are available upon request.

## References

[CR1] Abhilash PC, Yunus M (2011). Can we use biomass produced from phytoremediation?. Biomass Bioenerg.

[CR2] Abubakar MM, Anka US, Ahmad MM, Getso BU (2014). The potential of *Amaranthus caudatus* as a phytoremediating agent for lead. J Environ Earth Sci.

[CR3] Adefemi OS, Ibigbami OA, Awokunmi EE (2012). Level of heavy metals in some edible plants collected from selected dumpsites in Ekiti State, Nigeria. Glob Adv Res J Environ Sci Toxicol.

[CR4] Adefila EO, Onwordi CT, Ogunwande IA (2010). Level of heavy metals uptake on vegetables planted on poultry droppings dumpsite. Arch Appl Sci Res.

[CR5] Adewuyi GO, Dawodu FA, Jibiri NN (2010). Studies of the concentration levels of heavy metals in vegetable (*Amaranthus caudatus*) grown in dumpsites within Lagos Metropolis, Nigeria. Pac J Sci Tech.

[CR6] Aery NC, Rana DK (2007). Interactive effects of Zn, Pb and Cd in barley. J Environ Sci Eng.

[CR7] Aghelan N, Sobhanardakani S, Cheraghi M, Lorestani B, Merrikhpour H (2021). Evaluation of some chelating agents on phytoremediation efficiency of *Amaranthus*
*caudatus* L. and *Tagetes*
*patula* L. in soils contaminated with lead. J Environ Health Sci.

[CR8] Agoramoorthy G, Chen F, Venkatesalu V, Shea P (2009). Bioconcentration of heavy metals in selected medicinal plants of India. J Environ Biol.

[CR9] Aguilera-Cauich EA, Solís-Fernández KZ, Ibarra-Morales A, Cifuentes-Velásquez R, Sánchez del-Pino I (2021). Amaranth: distribution and morphological diversity of the genetic resource in parts of the Mayan region (southeast of Mexico, Guatemala and Honduras). Act Bot Mex.

[CR10] Ahmed S, Zohra FT, Mahdi MM, Nurnabi M, Alam MZ, Choudhury TR (2022). Health risk assessment for heavy metal accumulation in leafy vegetables grown on tannery effluent contaminated soil. Toxicol Rep.

[CR11] Akubugwo EI, Obasi A, Chinyere GC, Eze E, Nwokeoji O, Ugbogu EA (2012). Phytoaccumulation effects of *Amaranthus*
*hybridus* L. grown on Buwaya refuse dumpsites in Chikun, Nigeria on heavy metals. J Biodiv Environ Sci.

[CR12] Aladesanmi OT, Oroboade JG, Osisiogu CP, Osewole AO (2019). Bioaccumulation factor of selected heavy metals in *Zea mays*. J Health Pollut.

[CR13] Ali H, Khan E, Sajad MA (2013). Phytoremediation of heavy metals—concepts and applications. Chemosphere.

[CR14] Alizadeh A, Ghorbani J, Motamedi J, Vahabzadeh G, Edraki M, van der Ent A (2021). Metal and metalloid accumulation in native plants around a copper mine site: implications for phytostabilization. Int J Phytoremediat.

[CR15] Alsherif EA, Al-Shaikh TM, AbdElgawad H (2022). Heavy metal effects on biodiversity and stress responses of plants inhabiting contaminated soil in Khulais, Saudi Arabia. Biology.

[CR16] Amin H, Arain BA, Jahangir TM, Abbasi MS, Amin F (2018). Accumulation and distribution of lead (Pb) in plant tissues of guar (*Cyamopsis*
*tetragonoloba* L.) and sesame (*Sesamum*
*indicum* L.): profitable phytoremediation with biofuel crops. Geol Ecol Landsc.

[CR17] Antonkiewicz J, Jasiewicz C (2002). The use of plants accumulating heavy metals for detoxification of chemically polluted soils. Electron J Pol Agric Univ.

[CR18] Armas C, Ordiales R, Pugnaire FI (2004). Measuring plant interactions: a new comparative index. Ecology.

[CR19] Atayase MO, Eigbadon AI, Oluwa KA, Adesodun JK (2009). Heavy metal contamination of *Amaranthus* grown along major highways in Lagos, Nigeria. Afr Crop Sci J.

[CR20] Baldantoni D, Morra L, Zaccardelli M, Alfani A (2016). Cadmium accumulation in leaves of leafy vegetables. Ecotoxicol Environ Saf.

[CR21] Bech J (2022). Soil contamination and human health: recent contributions. Environ Geochem Health.

[CR22] Berton RS, Pires AMM, Andrade SALD, Abreu CAD, Ambrosano EJ, Silveira APDD (2006). Nickel toxicity in common bean plants and effects on soil microbiota. Pesqui Agropecu Bras.

[CR23] Boamponsem GA, Kumi M, Debrah I (2012). Heavy metals accumulation in cabbage, lettuce and carrot irrigated with wastewater from Nagodi mining site in Ghana. Int J Sci Technol Res.

[CR24] Bosiacki M, Kleiber T, Kaczmarek J (2013). Evaluation of suitability of *Amaranthus*
*caudatus* L. and *Ricinus*
*communis* L. in phytoextraction of cadmium and lead from contaminated substrates. Arch Environ Prot.

[CR25] Briffa J, Sinagra E, Blundell R (2020). Heavy metal pollution in the environment and their toxicological effects on humans. Heliyon.

[CR26] Canty A, Ripley B (2015) boot: Bootstrap R (S-Plus) functions. R package version 1. 3–16. Retrieved from https://cran.r-project.org/web/packages/boot/

[CR27] Carrión CS, Mendoza WJ (2019). Potential phytoremediator of native species in soils contaminated by heavy metals in the garbage dump Quitasol-Imponeda Abancay. J Sustain Dev Energy Water Environ.

[CR28] Chandrasekhar C, Ray JG (2019). Lead accumulation, growth responses and biochemical changes of three plant species exposed to soil amended with different concentrations of lead nitrate. Ecotoxicol Environ Saf.

[CR29] Chi K, Zou R, Wang L, Huo W, Fan H (2019). Cellular distribution of cadmium in two amaranth (*Amaranthus*
*mangostanus* L.) cultivars differing in cadmium accumulation. Environ Sci Pollut Res.

[CR30] Chinmayee MD, Mahesh B, Pradesh S, Mini I, Swapna TS (2012). The assessment of phytoremediation potential of invasive weed *Amaranthus spinosus* L.. Appl Biochem Biotechnol.

[CR31] Chunilall V, Kindness A, Jonnalagadda SB (2005). Heavy metal uptake by two edible *Amaranthus* herbs grown on soils contaminated with lead, mercury, cadmium, and nickel. J Environ Sci Health B.

[CR32] Cui X, Mao P, Sun S, Huang R, Fan Y, Li Y, Li Y, Zhuang P, Li Z (2021). Phytoremediation of cadmium contaminated soils by *Amaranthus*
*hypochondriacus* L.: The effects of soil properties highlighting cation exchange capacity. Chemosphere.

[CR33] Davison AC, Hinkley DV (1997) Bootstrap methods and their application. Technometrics 94(445). 10.1017/CBO9780511802843

[CR34] Devlaeminck R, Bossuyt B, Hermy M (2004). The effect of sampling period on results of seedling germination experiments in cropland and forests. Belg J Bot.

[CR35] Ding P, Zhuang P, Li Z, Xia H, Lu H (2013). Accumulation and detoxification of cadmium by larvae of *Prodenia litura* (Lepidoptera: Noctuidae) feeding on Cd-enriched amaranth leaves. Chemosphere.

[CR36] Egwu OC, Casmir UC, Victor UC, Samuel UC, Dickson MA, Oluwanisola OW (2019). Evaluation and ecological risk assessment of selected heavy metal pollution of soils and *Amaranthus cruentus* and *Telfairia occidentalis* grown around dump site in Chanchaga Minna, Niger State, Nigeria. Asian J Environ Ecol.

[CR37] El-Amier YA, Alghanem SM, Alzuaibr FM (2017). Bioaccumulation and translocation of heavy metals from coastal soil by wild halophytes. Am J Environ Prot.

[CR38] Eze MO (2014). Heavy metal concentrations in soils and accumulation in *Amaranthus hybridus* grown near solid waste dumpsites in Gombe, Nigeria. Int J Curr Res.

[CR39] Fan HL, Zhou W (2009). Screening of amaranth cultivars (*Amaranthus mangostanus* L.) for cadmium hyperaccumulation. Agr Sci China.

[CR40] FAO (2018) Soil pollution: a hidden reality. Food & Agriculture Organization

[CR41] Freitas MA, Medeiros FHV, Carvalho SP, Guilherme LRG, Teixeira WD, Zhang H, Paré PW (2015). Augmenting iron accumulation in cassava by the beneficial soil bacterium *Bacillus subtilis* (GBO3). Front Plant Sci.

[CR42] Gall JE, Boyd RS, Rajakaruna N (2015). Transfer of heavy metals through terrestrial food webs: a review. Environ Monit Assess.

[CR43] Garba MDM, Kiyawa SA (2018). Heavy metals accumulation in amaranths (*Amaranthus hybridus*) and jews mallow (*Corchorus olitorius*) grown on soil amended with tannery sludge from Challawa industrial area, Kano State, Nigeria. IOSR J Environ Sci Toxicol Food Techn.

[CR44] Ghazaryan KA, Movsesyan HS, Minkina TM, Sushkova SN, Rajput VD (2021). The identification of phytoextraction potential of *Melilotus officinalis* and *Amaranthus retroflexus* growing on copper- and molybdenum-polluted soils. Environ Geochem Health.

[CR45] Hauptvogl M, Kotrla M, Prčík M, Pauková Ž, Kováčik M, Lošák T (2020). Phytoremediation potential of fast-growing energy plants: challenges and perspectives – a review. Pol J Environ Stud.

[CR46] Hesami R, Salimi A, Ghaderian SM (2018). Lead, zinc, and cadmium uptake, accumulation, and phytoremediation by plants growing around Tang-e Douzan lead–zinc mine, Iran. Environ Sci Pollut Res.

[CR47] Huang Y, Xi Y, Gan L, Johnson D, Wu Y, Ren D, Liu H (2019). Effects of lead and cadmium on photosynthesis in *Amaranthus spinosus* and assessment of phytoremediation potential. Int J Phytoremediat.

[CR48] Huang X, Duan S, Wu Q, Yu M, Shabala S (2020). Reducing cadmium accumulation in plants: structure-function relations and tissue-specific operation of transporters in the spotlight. Plants (Basel).

[CR49] Iamonico D (2020). Nomenclature survey of the genus *Amaranthus* (*Amaranthaceae*). 10. What is *Amaranthus*
*commutatus*?. Thaiszia J Bot Košice.

[CR50] Khoramnejadian S, Saeb K (2018). Accumulation and translocation of heavy metals by *Amaranthus retroflexus*. J Earth Environ Health Sci.

[CR51] Ko CH, Chang FC, Wang YN, Chung CY (2013). Extraction of heavy metals from contaminated soil by two *Amaranthus* spp. CLEAN - Soil Air Water.

[CR52] Lago-Vila M, Arenas-Lago D, Rodríguez-Seijo A, Andrade Couce ML, Vega FA (2015). Cobalt, chromium and nickel contents in soils and plants from a serpentinite quarry. Solid Earth.

[CR53] Liu N, Dai J, Tian H, He H, Zhu Y (2019). Effect of ethylenediaminetetraacetic acid and biochar on Cu accumulation and subcellular partitioning in *Amaranthus retroflexus* L. Environ Sci Pollut Res.

[CR54] Liu C, Xiao R, Dai W, Huang F, Yang X (2021). Cadmium accumulation and physiological response of *Amaranthus*
*tricolor* L. under soil and atmospheric stresses. Environ Sci Pollut Res.

[CR55] Lombi E, Zhao F, Dunham S, McGrath S (2001). Phytoremediation of heavy metal-contaminated soils: natural hyperaccumulation versus chemically enhanced phytoextraction. J Environ Qual.

[CR56] Lu Y, Yao H, Shan D, Jiang Y, Zhang S, Yang J (2015). Heavy metal residues in soil and accumulation in maize at long-term wastewater irrigation area in Tongliao, China. J Chem-NY.

[CR57] Lukatkin AS, Bashmakov DI, Harbawee WEQA, Teixeira da Silva JA (2021). Assessment of physiological and biochemical responses of *Amaranthus retroflexus* seedlings to the accumulation of heavy metals with regards to phytoremediation potential. Int J Phytoremediat.

[CR58] Mellem JJ, Baijnath H, Odhav B (2012). Bioaccumulation of Cr, Hg, As, Pb, Cu and Ni with the ability for hyperaccumulation by *Amaranthus dubius*. Afr J Agric Res.

[CR59] Melo GW, Furini G, Brunetto G, Comin JJ, Guimarães Simão D, Marques ACR, Marchezan C, Silva ICB, Souza M, Soares CR, Zalamena J (2022). Identification and phytoremediation potential of spontaneous species in vineyard soils contaminated with copper. Int J Phytoremediat.

[CR60] Motesharezadeh B, Savaghebi-Firoozabadi GR, Mirseyed H, Alikhani HA (2010). Study of the enhanced phytoextraction of cadmium in a calcareous soil. Int J Environ Res.

[CR61] Nejatzadeh-Barandozi F, Gholami-Borujeni F (2014). Effectiveness of phytoremediation technologies to clean up of metalloids using three plant species in Iran. Water Environ Res.

[CR62] Ng JC, Juhasz A, Smith E, Naidu R (2015). Assessing the bioavailability and bioaccessibility of metals and metalloids. Environ Sci Pollut Res.

[CR63] Ogbenna CV, Yahaya AG, Nathaniel F (2019). Zinc accumulation in *Amaranthus caudatus* and *Corchorus olitorius*: relevance for phytoextraction. Asian J Adv Agric Res.

[CR64] Ogunkunle CO, Fatoba PO, Awotoye OO, Olorunmaiye KS (2013). Root-shoot partitioning of copper, chromium and zinc in *Lycopersicon esculentum* and *Amaranthus hybridus* grown in cement-polluted soil. Environ Exp Biol.

[CR65] Oladejo N, Anegbe B, Adeniyi O (2017). Accumulation of heavy metals in soil and maize plant (*Zea mays*) in the vicinity of two government approved dumpsites in Benin City, Nigeria. Asian J Chem Sci.

[CR66] Oluwatosin GA, Adeoyolanu OD, Ojo AO, Are KS, Dauda TO, Aduramigba-Modupe VO (2010). Heavy metal uptake and accumulation by edible leafy vegetable (*Amaranthus hybridus* L.) grown on urban valley bottom soils in Southwestern Nigeria. Soil Sediment Contam.

[CR67] Oti WJO (2015). Bioaccumulation factors and pollution indices of heavy metals in selected fruits and vegetables from a derelict mine and their associated health impacts. Int J Environ Sci Toxicol Res.

[CR68] Panagos P, van Liedekerke M, Yigini Y, Montanarella L (2013). Contaminated sites in Europe: review of the current situation based on data collected through a European Network. J Environ Pub Health.

[CR69] Prudent P, Ndong RO, Mebale AJA, Vassalo L, Demelas C, Mewono L, Ondo JA (2014). Metal accumulation in *Amaranthus cruentus* cultivated on different systems of tropical urban gardens. J Acad Indus Res.

[CR70] R Development Core and Team (2011) A language and environment for statistical computing. R Foundation for Statistical Computing, Vienna. http://www.R-project.org

[CR71] Rahman MM, Azirun SM, Boyce AN (2013). Enhanced accumulation of copper and lead in amaranth (*Amaranthus paniculatus*), Indian mustard (*Brassica juncea*) and sunflower (*Helianthus annuus*). Plos One.

[CR72] Rai PK, Lee SS, Zhang M, Tsang YF, Kim KH (2019). Heavy metals in food crops: health risks, fate, mechanisms, and management. Environ Int.

[CR73] Rajakaruna N, Boyd RS, Harris TB (eds) (2014) Plant ecology and evolution in harsh environments (environmental research advances), Nova Science Pub Inc., UK ed

[CR74] Ramanlal DB, Kumar RN, Kumar N, Thakkar R (2020). Assessing potential of weeds (*Acalypha indica* and *Amaranthus viridis*) in phytoremediating soil contaminated with heavy metals-rich effluent. SN Appl Sci.

[CR75] Ramírez A, Garcia G, Werner O, Navarro-Pedreño J, Ros RM (2021). Implications of the phytoremediation of heavy metal contamination of soils and wild plants in the industrial area of Haina. Dominican Republic Sustain.

[CR76] Ranđelović D, Jakovljević K, Mihailović N, Jovanović S (2018). Metal accumulation in populations of *Calamagrostis*
*epigejos* (L.) Roth from diverse anthropogenically degraded sites (SE Europe, Serbia). Environ Monit Assess.

[CR77] Rehman ZU, Khan S, Shah MT, Brusseau ML, Khan SA, Mainhagu J (2018). Transfer of heavy metals from soils to vegetables and associated human health risks at selected sites in Pakistan. Pedosphere.

[CR78] Roveda LF, Cuquel FL, Motta ACV, Melo VF (2016). Organic compounds with high Ni content: effects on soil and strawberry production. Rev Bras Eng Agr Amb.

[CR79] Shagal MH, Maina HM, Donatus RB, Tadzabia K (2012). Bioaccumulation of trace metals concentration in some vegetables grown near refuse and effluent dumpsites along Rumude-Doubeli bye-pass in Yola North, Adamawa State. Glob Adv Res J Environ Sci Toxicol.

[CR80] Shang E, Xu E, Zhang H, Huang C (2019). Temporal-spatial trends in potentially toxic trace element pollution in farmland soil in the major grain-producing regions of China. Sci Rep-UK.

[CR81] Sharma NC, Gardea-Torresdey JL, Parsons J, Sahi SV (2004). Chemical speciation and cellular deposition of lead in *Sesbania drummondii*. Environ Toxicol Chem.

[CR82] Sharma S, Tiwari S, Hasan A, Saxena V, Pandey LM (2018). Recent advances in conventional and contemporary methods for remediation of heavy metal-contaminated soils. 3 Biotech.

[CR83] Shojaei S, Jafarpour A, Shojaei S, Gyasi-Agyei Y, Rodrigo-Comino J (2021). Heavy metal uptake by plants from wastewater of different pulp concentrations and contaminated soils. J Cleaner Prod.

[CR84] Suman J, Uhlik O, Viktorova J, Macek T (2018). Phytoextraction of heavy metals: a promising tool for clean-up of polluted environment?. Front Plant Sci.

[CR85] Tőzsér D, Magura T, Simon E (2017). Heavy metal uptake by plant parts of willow species: a meta-analysis. J Hazard Mater.

[CR86] Tőzsér D, Tóthmérész B, Harangi S, Baranyai E, Lakatos G, Fülöp Z, Simon E (2019). Remediation potential of early successional pioneer species *Chenopodium album* and *Tripleurospermum inodorum*. Nat Conserv-Bulgaria.

[CR87] Ullah S, Khan J, Hayat K, Elateeq AA, Salam U, Yu B, Ma Y, Wang H, Tang ZH (2020). Comparative study of growth, cadmium accumulation and tolerance of three chickpea (*Cicer*
*arietinum* L) cultivars. Plants.

[CR88] Wadgaonkar SL, Ferraro A, Nancharaiah YV, Dhillon KS, Fabbricino M, Esposito G, Lens PNL (2019). In situ and ex situ bioremediation of seleniferous soils from northwestern India. J Soils Sediment.

[CR89] Wolosik K, Markowska A (2019). *Amaranthus cruentus* taxonomy, botanical description, and review of its seed chemical composition. Nat Prod Commun.

[CR90] Xu D, Shen Z, Dou C, Dou Z, Li Y, Gao Y, Sun Q (2022). Effects of soil properties on heavy metal bioavailability and accumulation in crop grains under different farmland use patterns. Sci Rep.

[CR91] Yan A, Wang Y, Tan SN, Yusof MLM, Ghosh S, Chen Z (2020). Phytoremediation: a promising approach for revegetation of heavy metal-polluted land. Front Plant Sci.

[CR92] Yang W, Wang Y, Liu D, Hussain B, Ding Z, Zhao F, Yang X (2020). Interactions between cadmium and zinc in uptake, accumulation and bioavailability for *Salix integra* with respect to phytoremediation. Int J Phytoremediat.

[CR93] Yap CK, Yaacob A, Tan WS, Al-Mutairi KA, Cheng WH, Wong KW, Edward FE, Ismail MS, You CF, Chew W, Nulit R, Ibrahim MH, Amin B, Sharifinia M (2022). Potentially toxic metals in the high-biomass non-hyperaccumulating plant *Amaranthus viridis*: human health risks and phytoremediation potentials. Biology.

[CR94] Ziarati P, Somaye Alaedini PZ (2014). The phytoremediation technique for cleaning up contaminated soil by *Amaranthus* sp. J Environ Anal Toxicol.

[CR95] Zou J, Wang M, Jiang W, Liu D (2006). Chromium accumulation and its effects on other mineral elements in *Amaranthus viridis* L.. Acta Biol Cracov Bot.

