# Supplementary material for: A meta-analysis on the heavy metal uptake in Amaranthus species
Source: Environ Sci Pollut Res Int. 2023 Jun 28;30(36):85102–12. doi: 10.1007/s11356-023-28374-3 (PMC10404196; doi:10.1007/s11356-023-28374-3)
Supplement: Supplementary file 1 — Supplementary file1 (DOCX 412 KB) [file 11356_2023_28374_MOESM1_ESM.docx]

Supplementary Information Table 1. Pictures and sources of studied Amaranthus species.

|  | Name | Sources | Citation |
| --- | --- | --- | --- |
| 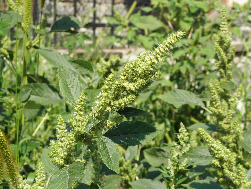 | *Amaranthus retroflexus L.* | https://powo.science.kew.org/taxon/urn:lsid:ipni.org:names:10698-2 | Alsherif et al. 2022, Ghazaryan et al. 2019, Khoramnejadian & Saeb 2015, Liu et al. 2019, Motesharezadeh et. al. 2010, Nejatzadeh-Barandozi & Gholami-Borujeni 2014 |
| 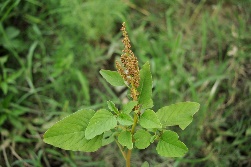 | *Amaranthus viridis L.* | https://powo.science.kew.org/taxon/urn:lsid:ipni.org:names:316349-2 | Atayase et al. 2008, Zou et al. 2006 |
| 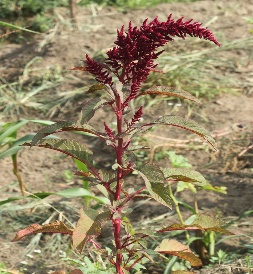 | *Amaranthus caudatus L.* | https://powo.science.kew.org/taxon/urn:lsid:ipni.org:names:316347-2 | Bosiacki et al. 2013, |
| 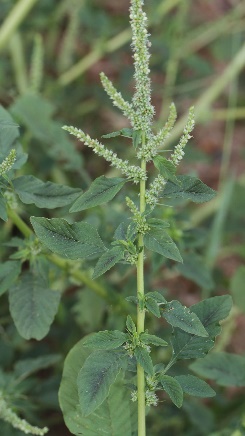 | *Amaranthus spinosus L.* | https://powo.science.kew.org/taxon/urn:lsid:ipni.org:names:10711-2 | Chinmayee et al. 2012, Huang et al. 2019, |
| 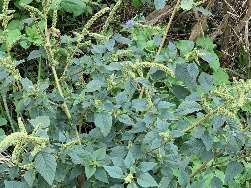 | *Amaranthus dubius Mart. ex Thell.*  This name is a synonym of *Amaranthus tortuosus* | https://powo.science.kew.org/taxon/urn:lsid:ipni.org:names:59693-1 | Chunilall et al. 2005, Ramírez et al. 2021 |
| 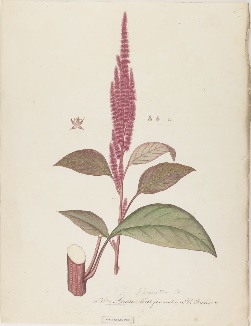 | *Amaranthus hybridus Vell.*  This name is a synonym of *Amaranthus hypochondriacus* | https://powo.science.kew.org/taxon/urn:lsid:ipni.org:names:10665-2 | Chunilall et al. 2005, Cui et al. 2021, Ding et al. 2013, Eze 2014, Garba & Kijawa 2018, |
| 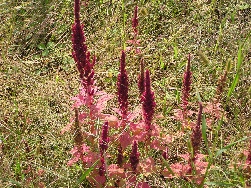 | *Amaranthus cruentus L.* | https://powo.science.kew.org/results?q=Amaranthus%20cruentus | Egwu et al. 2019 |
| 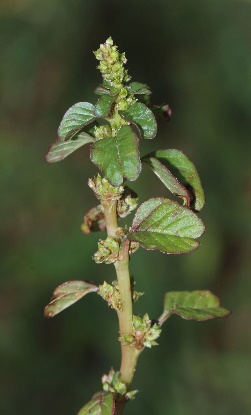 | *Amaranthus tricolor L.* | https://powo.science.kew.org/taxon/urn:lsid:ipni.org:names:327386-2 | Liu et al. 2021 |
